# Clinic Attendance for Medication Refills and Medication Adherence amongst an Antiretroviral Treatment Cohort in Uganda: A Prospective Study

**DOI:** 10.1155/2010/872396

**Published:** 2010-12-19

**Authors:** Setor Kunutsor, John Walley, Elly Katabira, Simon Muchuro, Hudson Balidawa, Elizabeth Namagala, Eric Ikoona

**Affiliations:** ^1^Nuffield Centre for International Health and Development, Institute of Health Sciences, Leeds University, Leeds LS2 9LJ, UK; ^2^Department of Medicine, Makerere University College of Health Sciences, Kampala, Uganda; ^3^Uganda National Aids Control Programme, Uganda

## Abstract

*Background*. Regular clinic attendance for antiretroviral (ARV) drug refills is important for successful clinical outcomes in HIV management. *Methods*. Clinic attendance for ARV drug refills and medication adherence using a clinic-based pill count in 392 adult patients receiving antiretroviral therapy (ART) in a district hospital in Uganda were prospectively monitored over a 28-week period. *Results*. Of the 2267 total scheduled clinic visits, 40 (1.8%) were missed visits. Among the 392 clients, 361 (92%) attended all appointments for their refills (regular attendance). Clinic attendance for refills was statistically significantly associated with medication adherence with regular attendant clients having about fourfold greater odds of achieving optimal (≥95%) medication adherence [odds ratio (OR) = 3.89, 95% CI: 1.48 to 10.25, exact *P* = .013]. In multivariate analysis, clients in age category 35 years and below were less likely to achieve regular clinic attendance. *Conclusion*. Monitoring of clinic attendance may be an objective and effective measure and could be a useful adjunct to an adherence measure such as pill counting in resource-constrained settings. Where human resource constraints do not allow pill counts or other time-consuming measures, then monitoring clinic attendance and acting on missed appointments may be an effective proxy measure.

## 1. Introduction

HIV affects an estimated 33 million worldwide with approximately 22 million of these in subSaharan Africa [1]. HIV prevalence is estimated at 5–15% in most subSaharan African countries with Uganda having an estimated prevalence of 5.4% among adults [1]. The widespread use of ARVs in the treatment of HIV has led to significant decreases in HIV-related morbidity and mortality worldwide [2]. Access to ART has been scaled up throughout the country since the rollout of free ART in Uganda in 2004 [3]. There are currently 336 accredited ART sites across the country and about 190,000 people are now accessing ART countrywide [4].

Nonadherence is the most common cause of treatment failure and the development of resistance to ARVs [5]. Though factors such as antiretroviral regimen potency and medication adherence all play a role in the development of antiretroviral medication resistance [6], the goal of HIV therapy is to achieve near-perfect adherence leading to sustained virologic suppression [7]. As more people gain access to ART, there is the need to find simple, efficient and replicable ways of assessing and maintaining these near-perfect levels of adherence to ensure sustained virologic suppression. 

Adherence to ART is measured by a variety of methods with the most commonly used methods being self-reporting by patients, pill counts, review of pharmacy records and Medication Event Monitoring System (MEMS) caps [8]. There are also biochemical measures, such as viral loads and therapeutic drug monitoring. All these methods have their shortcomings. No gold standard has as yet been found for evaluating adherence to medication to date [9]. Measuring medication adherence in clinical settings has not always been easy because common methods such as pill counts are not objective enough and the more accurate measures of adherence such as drug-level monitoring are expensive, can be inconvenient, difficult to implement in resource poor settings, and not very relevant since it can only practically be done intermittently and appears to be only sensitive for identifying very low levels of adherence [10]. However, patients' clinic attendance for medication refills can easily be monitored by the health staff, even in resource-constrained settings [11, 12]. Inconsistent attendance may help identify patients in need of adherence counselling [13]. In resource-rich countries, poor clinic attendance has been shown to be correlated with mortality [9]. Missed appointments have also been shown to be a strong predictor for virological failure [14, 15]. The International Network for the Rational Use of Drugs Initiative on Adherence to Antiretroviral Therapy (INRUD-IAA) [16] has recently identified, tested and validated against clinical outcomes, core indicators for adherence to ARV medicines, and treatment defaulting, one of which includes patient attendance at appointments [17]. Consistent or regular clinic attendance plays a key role in prolonging life and enhancing quality of life for people living with HIV/AIDS [18].

Few studies have assessed ART adherence based on clinic attendance [17, 19]. Previous studies have shown that clinic attendance is associated with medication adherence in patients with chronic illness [20, 21]. Appointment attendance has also been often used as a proxy for medication adherence in nonHIV/AIDS treatment settings [22, 23]. Most of the little published data evaluating the medication adherence—clinic attendance relationship in HIV patients on ART have been from cross-sectional surveys and retrospective collection of data. A cross-sectional questionnaire survey conducted in India reported a statistically significant relationship between 100% medication adherence and regular followup attendance [24]. Giordano and colleagues [25] in their retrospective cohort study reported a significant relationship between regular clinic attendance or retention in care and antiretroviral medication adherence. In another retrospective cohort study of 16 HIV/AIDS treatment facilities in the eastern part of Africa, it was demonstrated that dispensing-based adherence, self-reported adherence, and consistent visit attendance were significantly correlated [17]. The relationships reported have been as a result of brief cross-sectional measurements and extraction of data from records, therefore they do not provide enough information about the longitudinal relationship between clinic attendance and ART adherence.

In our prospective study, we set out to assess clinic attendance for medication refills amongst an antiretroviral treatment cohort, determine the association between clinic attendance and adherence to ART and assess the factors associated with optimal medication adherence and regular clinic attendance.

## 2. Materials and Methods

### 2.1. Study Area

The study was conducted in Kayunga Hospital located in the Kayunga District of central Uganda. Kayunga hospital is a 123 bed capacity health facility located in a rural setting. It receives ARVs from Makerere University Walter Reed Project (MUWRP) and the Uganda Ministry of Health. This site is a government accredited ART site and provides ARVs and ART care free of charge. There are four health centres in the district that also provide ARVs and ART care free of charge and with support from the MUWRP. In the Kayunga Hospital HIV/ART clinic like in all accredited ART sites in Uganda, patients before initiating ART are usually required to in addition to other criteria, disclose their HIV status to a chosen friend or family member. Such an individual is known as a treatment supporter and should be chosen in discussion with the patient, have the patient's trust, should be available to go to the preparatory visits with the patient, and should be committed to support the patient with ART over a long time.

### 2.2. Study Design and Participants

A prospective cohort design was employed for this study. It was part of a project by the Communicable Disease Research Programme Consortium (COMDIS) in Uganda to identify feasible and replicable methods of improving medication adherence for use in the context of resource-constrained settings. It was conducted under the routine operational programme conditions of the HIV/ART clinic and was implemented with minimum additional inputs, reflecting real-life settings. The study population in this study included adults (18 years and over) who were receiving treatment at the Kayunga Hospital HIV/ART clinic. All adult patients visiting the clinic and had been receiving ART for at least three months as at the beginning of the study period were agreeable to be part of the study and were included. The majority of patients were receiving a first-line fixed dose combination regimen consisting of Zidovudine, Lamivudine and Nevirapine or Stavudine, Lamivudine and Nevirapine. Clinic attendance for their four-weekly refill of ARVs was monitored for this cohort over a 28-week period. During the study period, patients received regular health education sessions and adherence counselling support which was part of the routine care that the HIV/ART clinic provided and are typical for Uganda and other HIV/AIDS treatment programmes in subSaharan Africa. The ethics committee of the Makerere University Medical School reviewed and approved the study procedures and data collection instruments. Verbal and written consents were obtained for all participants.

### 2.3. Data Collection

Trained adherence workers and research assistants, all locals in the study area, were used for data collection. Data was collected on the clients' sociodemographic profile, type of ART combination regimen and duration on ART. Clinic attendance for each client was monitored every four weeks based on the given clinic appointment dates for ARV refills (these were scheduled at four-weekly intervals). Clinic attendance was categorised into “regular attendance” and “inconsistent attendance”. Regular clinic attendance was defined as the patient visiting the clinic for their refills on the scheduled appointment dates for the entire study period (also included clients who attended appointments before the day scheduled or those who were late by about three days and had extra pills to cover this period). “Inconsistent attendance” was defined as missing one or more clinic appointments (but not consecutive) for refills during the study period. A medication adherence assessment was also conducted at the same time on each client who reported at the health facility for their four-weekly refill of ARTs using a clinic-based pill count. Medication adherence was defined as the percentage of prescribed medication doses taken during each 4-week period [26] with levels of 95% and over being categorised as “optimal” and levels less than 95% as “suboptimal”. Adherence using pill count was calculated by dividing the number of pills actually taken by the number of pills the client was required to take over the reporting period multiplied by 100. Full details of how adherence assessment was conducted have been reported elsewhere [3]. Clients who missed visits were followed-up and reasons for their missed visits were ascertained. Data was also collected on clients who were transferred out, lost to follow up or died during study period. Deaths reported were the ones confirmed by the clinic after followup. Those lost to followup potentially included deaths but which could not be confirmed because their whereabouts could not be traced during followup. Loss to followup was defined as missing more than two consecutive clinic appointments after the date of last attendance [27, 28]. Transferred out was recorded for patients who formally transferred to another treatment centre. Zero medication adherence was awarded to patients who missed visits and were known not to have collected medications from the pharmacy and those patients who were incapacitated or sick and were known not to have taken their medications.

### 2.4. Statistical Analysis

Data were entered and analysed using Statistical Package for the Social Sciences (SPSS) version 17.0 for windows (SPSS Inc., Chicago, USA). Descriptive analysis was employed for the basic demographic, ART treatment characteristics and clinic attendance categories. Individual mean medication adherence levels using pill counts were determined for the period of data collection. The mean medication adherence for the study sample and proportions of clients achieving adherence levels of 95% and over were also determined and 95% confidence intervals (CIs) calculated. Fisher's exact test was used to determine the association between clinic attendance for refills and medication adherence. Factors associated with optimal adherence and regular clinic attendance were determined using multivariate logistic regression analyses. *P*-values <  .05 (two-sided) were considered statistically significant. All results were given as mean (standard deviation (SD)) unless indicated otherwise.

## 3. Results

### 3.1. Demographic Data and Patient Characteristics

Baseline characteristics of the 392 study participants are shown in Table 1. Median age was 38 years with 65% of study participants being female. 73% of clients had only a primary education or less. The majority of clients were unemployed (55%) and had been on ART for more than 2 years (63%). All clients reported having a treatment supporter but we had no way of determining if this was still active.

### 3.2. Clinic Attendance Patterns

A total of 2267 clinic appointments were scheduled for the 392 clients during the 28-week study period with 31 clients missing 40 clinic visits. Clinic attendance patterns are shown in Table 2.

Self-reported major reasons cited for missed visits were travel and financial constraints because of the long distance involved in travelling to the clinic—36 (66.7%) and forgetfulness—12 (22.2%). Other reasons included being too ill to come to clinic—3 (5.6%); depression—1 (1.9%); commitments with work or other activities—1 (1.9%); and being discouraged from returning because of the long delays at the clinic—1 (1.9%)

### 3.3. Adherence Levels

Mean medication adherence (95% CI) was 98.4% (98.1%–98.6%) with 365 (93.1%) of study participants achieving optimal medication adherence.

### 3.4. Clinic Attendance and Adherence

Table 3 shows numbers of clients and their patterns of clinic attendance according to the optimal or suboptimal adherence categories. Of the 31/392 (7.9%) clients whose clinic attendance for refills were inconsistent, only 6/31 (19.4%) achieved suboptimal medication adherence for the entire duration of the study. 

A Fisher's exact test showed there was a significant association between clinic attendance for refills and medication adherence (exact *P* = .013). Clients with regular attendance were more likely to achieve optimal adherence compared to clients with inconsistent attendance: (OR = 3.89, 95% CI: 1.48 to 10.25); (risk ratio = 1.20, 95% CI: 0.98 to 1.47); (risk difference = 0.15, 95% CI: −0.01 to 0.31). A multivariate logistic regression analysis showed that clients whose visits were inconsistent and those who were not educated were significantly less likely to achieve optimal adherence (Table 4).

### 3.5. Predictors of Clinic Attendance

A multivariate logistic regression analysis showed that those in age category 35 years and below were significantly less likely to achieve regular attendance (Table 5). There were no other significant associations.

## 4. Discussion

Our findings show that there is a high level of regular clinic attendance amongst ART clients for their ARV refills in this resource-constrained setting. Of the 392 clients, 92% were fully attendant during the entire study period. This is despite our use of a more stringent definition of regular clinic attendance—a client attending all scheduled appointments during the entire study period. Other studies have used less stringent definitions such as a client attending greater than 90% of three monthly appointments [24]. Regular clinic attendance for ARV drugs refills is important for successful clinical outcomes in HIV treatment [29]. Park et al. [30] demonstrated that adherence to clinic visits was associated with clinical improvement regardless of clinical category when ART was started. Patients who attend clinic regularly for their refills also have the opportunity to be treated for HIV-related diseases. Timeliness for appointments has been demonstrated to be positively correlated with better outcomes amongst patients on ART [14, 15]. 

In our study, major reasons given for missed visits included travel and financial constraints and forgetfulness, which are consistent with findings reported in other studies [31, 32]. Other reasons included depression and commitments with work or other activities. Patients' social situation may have a significant impact on their ability to consistently attend their clinic appointments for refills. Attendance to clinic appointments will more often not be regular in an unstable social environment. There is the need to identify trusted family members, partners and friends as treatment supporters who will assist with patients' appointment keeping, provide them with emotional support and may sometimes represent them when they are too ill to attend the clinic for their refills. This is a requirement in all ART-accredited sites in Uganda before initiating clients on ART but some clients for fear of stigmatisation will prefer not to disclose their status. It has been reported that the number of missed visits are directly related to the rate of disease progression [30]. Missed appointments should trigger concerns about patients at risk of defaulting on their treatment. More feasible, practical, and sustainable ways of providing ART need to be developed to avoid the issue of missed appointments. Home-based HIV-care has been shown to be as effective as the facility-based care and could enable equitable access to HIV treatment, especially in areas with poor access to care [33]. Mobile clinics and the setting up of more ART treatment facilities which are closer to home will help reduce transportation problems and costs. 

Patients on ART treatment in this resource-constrained setting, take on average 98% of their prescribed ART with about 93% achieving optimal adherence. These high levels of ART adherence are broadly consistent with the results of other studies conducted in such resource-constrained setting [3, 34–37]. Our study demonstrated that clinic attendance for refills was significantly associated with medication adherence with regular clinic attendants being more likely to achieve optimal adherence. Respondents who missed clinic appointments for refills were less likely to achieve optimal medication adherence. HIV-infected patients who miss clinic visits may also miss taking their antiretroviral medications [38, 39]. It is possible that patients who always attend the clinic on their appointment dates for their refills may be the ones more likely to be motivated to be adherent to their medication regimen. Clients who were not educated were also less likely to achieve optimal adherence. There were no associations with other sociodemographic variables. Some studies have found significant associations between good adherence and male gender and older age [40, 41] but other studies have found increasing age in years as significantly associated with suboptimal adherence [42, 43]. Generally, the role of social and demographic characteristics such as race, gender, age and educational level as predictors of adherence has produced mostly inconsistent results [43, 44]. Adherence levels exhibit both intra and interindividual variation [45] and the majority of people will exhibit low adherence some of the time [44]. Our study demonstrated that clients under 35 years of age were less likely to be fully attendant which is not surprising, as younger patients are more likely to have higher nonattendance rates; a finding which has been reported in several studies [46, 47]. Male gender and patients with no education were also less likely to be fully attendant but these associations were not statistically significant. Data from several studies suggest that cultural and sociodemographic characteristics influence patients' attendance to clinic appointments. Israelski et al. [48] demonstrated that patients of older age and receiving a higher income were more likely to keep medical appointments. Catz and colleagues [18] also reported that greater outpatient appointment nonattendance was associated with younger age, minority status and lower perceived social support. There is the need for research to better understand the predictors of clinic attendance, and then develop and test effective interventions for all patients; with more intensive intervention in those subsequently found to miss an appointment (s). 

### 4.1. Study Strengths and Limitations

Though informative, our study had several limitations which merit consideration. We were not able to corroborate attendance and adherence monitoring using pill counts with more objective indicators such as immunological, virological or clinical outcomes because of financial constraints particularly the cost of additional laboratory monitoring in this setting. Pill counts are commonly used to measure medication in research, they have a higher level of accuracy compared to patient self-reports and can be conducted when the patient returns to the clinic/pharmacy to obtain a refill [49]. But like self-reports, pill counts also tend to overestimate adherence [50, 51], often due to “pill dumping” [52]. They are too time-consuming and computationally complex for most clinical purposes and like most other adherence measures, the main drawback is that ingestion is not confirmed. The clinic-based pill count measure though not very objective compared to Medication Event Monitoring System (MEMS) caps and HIV viral loads, it has been reported to be correlated with viral loads [26, 34, 53]. Farley and colleagues [11] also demonstrated that appointment keeping behaviour correlated with virologic response. There were potentially important variables such as income of respondents, distance from the clinic, partner HIV testing and status, stage of HIV disease and coexisting opportunistic infections, which could affect clinic attendance and adherence to medications but were not presented in our multivariate analyses because of the unavailability of such data. The data was derived from a single site and so may not be generalisable to the whole country or elsewhere in subSaharan Africa. Our sample size was small with a followup period of 28 weeks; larger studies and longer followup periods may be required to confirm our results. An additional limitation is that there was the possibility of a potentially biased study population; it may be that we collected data from a cohort of more highly motivated people with regular clinic attendance and high adherence who gained the most from ART. Despite the various limitations, our study replicated the typical conditions under which treatment and care is delivered in such resource-poor settings. The benefits observed in this study may therefore be replicated in other sites. During the observation period, antiretroviral medications were always in stock at the study site.

### 4.2. Conclusions

Regular clinical attendance for medication refills and optimal adherence to ART have a critical influence on outcomes of ART. Continuity of care is essential to ensure the maximum health benefits of ART. There is a need to develop interventions which will improve clinic attendance among clients. Similarly, there is the need to develop feasible, practical, reliable and inexpensive methods of contacting and recalling patients who miss clinic appointments. Good medication adherence does not only entail compliance to medications; it also includes attendance at scheduled appointments [54]. Clinic attendance monitoring may be combined with adherence measures and clinic outcomes monitoring which will improve the objectivity of adherence measurements. A simple and standard approach for recording attendance and identifying patients who miss appointments such as a clinic appointment log, can help health facilities identify the problems and develop the appropriate interventions to improve clinic attendance. This approach has been traditionally used in TB registers, where the right hand side of the register is for recording the scheduled appointment date, and then above this, the actual date of attendance. Monitoring of clinic attendance may be an objective and effective measure and could be a useful adjunct to adherence measures and monitoring of clinical outcomes. Where human resource constraints and large numbers of ART patients do not allow pill counts or other time consuming measures, then monitoring clinic attendance for refills and acting on missed appointments may be an effective proxy measure.

##  Conflict of Interests

We declare that we have no conflicts of interest.

##  Authors' Contributions 

J. Walley and E. Katabira were the principal investigators, wrote the protocol, were involved in study design, supervised implementation of the study, and reviewed the paper. H. Balidawa, E. Namagala, and E. Ikoona were involved in protocol development, study facilitation and reviewing the paper. S. Kunutsor and S. Muchuro were involved in implementation of the study, coordination of field activities and data collection. S. Kunutsor was involved in design of study, devised data collection tools, inputted data, did the data analysis and interpretation and wrote the first draft. All were involved in critical revision of the paper and approved the final version.

##  Role of the Funding Source 

The research team of the Communicable Disease Research Programme Consortium participated in the design; collection, analysis, and interpretation of the data; writing of the report; and the decision to submit the paper for publication. The corresponding author had full access to all the data in the study and had final responsibility for the decision to submit for publication.

## Figures and Tables

**Table 1:** Sociodemographic and treatment characteristics (*N* = 392).

Variables	*n* (%)	Median (IQR)
Age (years)		38.0 (32.0–45.0)
Sex		
Male	138 (35.2)	
Female	254 (64.8)	
Educational level		
No education	58 (14.8)	
Primary	228 (58.2)	
Secondary	99 (25.3)	
Tertiary	7 (1.8)	
Employment		
Unemployed	217 (55.4)	
Employed	175 (44.6)	
Marital status		
Single	155 (39.5)	
Married	197 (50.3)	
Divorced	32 (8.2)	
Unmarried partner	8 (2.0)	
Time on ARV drugs (years)		2.5 (1.6–3.7)
≤2	147 (37.5)	
>2	245 (62.5)	

IQR, Interquartile range

**Table 2:** Summary of clients' clinic attendance during study period (*N* = 392).

Clinic Attendance Patterns	*n* (%)
Attended all appointments for refills (regular clinic attendance)	361 (92.1)
Missed one visit	24 (6.1)
Missed two or more visits	7 (1.8)
Transferred out*	11 (2.8)
Lost to followup*	3 (0.8)
Died (confirmed)*	1 (0.3)

*All these clients also had one or more missed visits prior to the recorded outcome.

**Table 3:** Comparison of clinic attendance with adherence for the ART treatment cohort.

	Optimal adherence	Suboptimal adherence	Total
Regular clinic attendance	340	21	361
Inconsistent attendance	25	6	31

Total	365	27	392

**Table 4:** Multivariate logistic regression analysis to determine predictors of optimal adherence.

Variable	OR [95% CI]	*P*
Sex		
Female	1.00	
Male	0.75 [0.30–1.91]	.546
Age (years)		
Greater than 35	1.00	
Less than or equal to 35	0.75 [0.31–1.82]	.529
Education		
Educated	1.00	
No education	0.32 [0.12–0.85]	.022
Employment		
Employed	1.00	
Unemployed	1.87 [0.81–4.30]	.143
Duration on ARVs		
More than 2 years	1.00	
Less than or equal to 2 years	0.62 [0.27–1.41]	.253
Clinic attendance		
Regular attendance	1.00	
Inconsistent attendance	0.28 [0.10–0.79]	.016

OR: odds ratio

**Table 5:** Multivariate logistic regression analysis to determine predictors of regular clinic attendance.

Variable	OR [95% CI]	*P*
Sex		
Female	1.00	
Male	0.47 [0.20–1.08]	.075
Age (years)		
Greater than 35	1.00	
Less than or equal to 35	0.33 [0.15–0.74]	.007
Education		
Educated	1.00	
No education	0.46 [0.17–1.25]	.128
Employment		
Employed	1.00	
Unemployed	1.33 [0.62–2.84]	.461
Duration on ARVs		
More than 2 years	1.00	
Less than or equal to 2 years	1.89 [0.82–4.34]	.136
